# Research on the evaluation model of pilots’ in-flight stress state based on flight critical events

**DOI:** 10.3389/fphys.2025.1713035

**Published:** 2026-02-18

**Authors:** Lamei Shang, Manting Lu, Chunying Qian, Xiaoru Wanyan, Yubin Zhou, Shuang Liu

**Affiliations:** 1 Country Aviation Medical Engineering Research Center, Air Force Medical University, PLA, Beijing, China; 2 Country School of Aeronautic Science and Engineering, Beihang University, Beijing, China

**Keywords:** flight stress, critical event, heart rate variability, respiratory signa, ensemblelearning

## Abstract

**Background:**

Stress refers to the non - specific response that occurs in an organism when it is subjected to stressful stimuli. When fighter pilots execute high-stress missions, they are likely to experience stress responses. These responses may have a negative impact on the cognitive decision-making processes of the pilots, subsequently undermining the mission effectiveness. However, to date, there is a dearth of methodologies for evaluating the in-flight stress states of pilots. The aim of this research is to establish an assessment model by using the real physiological data of pilots in flight to predict their stress state during flight.

**Method:**

This study utilized the in - flight physiological data of 32 pilots aged between 22 and 30 years old. The data included electrocardiogram (ECG) data, respiratory data, and human body acceleration data. By analyzing the differences in the physiological characteristics of pilots under various stress conditions, sensitive physiological indicators corresponding to different stress states were screened out. Using electrocardiogram (ECG) and respiratory indices that are sensitive to stress changes as model inputs, a stress state assessment model was constructed via machine learning techniques. The performance of the model was evaluated using accuracy, sensitivity, specificity, and the F1 - score.

**Result:**

Screen out ten electrocardiogram (ECG) indicators that are sensitive to the stress level of pilots, which are Mean HR, MeanRR, SDNN, RMSSD, pNN50, pNN20, LF/HF, LFn, HFn and SD1/SD2. Screen out five respiratory indicators that are sensitive to the stress level of pilots, which are Mean Rsp, EB1, EB2, EB3 and EB4. The binary classification assessment model of stress state, which was constructed using the ensemble learning approach, achieved an accuracy of 95.56% in the five - fold cross - validation.

**Conclusion:**

This study effectively accomplished the assessment of pilots’ stress states based on physiological characteristics. It is conducive to optimizing the stress training strategies for pilots and enhancing their capabilities to cope with and manage stress. This research has certain theoretical significance and engineering application value for improving the performance of flight missions and ensuring flight safety.

## 1 Introduction

Stress refers to the nonspecific response that occurs in an organism when it is exposed to stressful stimuli (Selye, 1950). In the realm of aviation, when carrying out missions, pilots are confronted with a complex and dynamic operational environment. Multiple aspects of flight situation changes, including mechanical malfunctions, meteorological variations, and the urgency of tasks, may induce stress responses of varying degrees in pilots (Hu, 2024). Numerous studies have demonstrated that alterations in stress levels can exert profound influences on pilots’ attentional resource allocation, flight task execution capabilities, and decision - making aptitudes (McClernon et al., 2011). Consequently, mitigating the adverse effects of an individual’s stress state on flight performance and safeguarding the operational safety and efficiency of personnel in complex and high - risk environments hold substantial significance within the domain of aviation safety research.

Studies have indicated that during certain crucial flight phases, the stress levels of pilots exhibit marked variations (Wang and Yang, 2024). Research conducted by Hormeño-Holgado has revealed that following the execution of offensive and defensive flight maneuvers in real - world flight scenarios, fighter pilots exhibit a significant increase in subjective stress perception and scores of perceived exertion rate. Moreover, their average heart rate rises significantly during the flight (Hormeño-Holgado and Clemente-Suárez, 2019). Fuentes-Garcia investigated the anxiety, perceived physical exertion, changes in self-confidence and autonomic nervous system responses of pilots during simulated and actual flights. The results indicated that actual flights significantly increased the RPE scores and average heart rates of the pilots (Fuentes-García et al., 2021). Johannes synthesized the psychophysiological arousal value by using multi-dimensional heart rate variability indicators to represent the psychophysiological arousal level of the operators, and evaluated the changes in the psychophysiological arousal level of pilots at different flight stages, such as takeoff, in-air refueling, and landing. The results show that the pilots’ arousal levels in the simulated flight scenarios were generally lower than those in the real flight scenarios, and they exhibited the highest arousal levels during the most challenging stage of in-flight refueling (Johannes et al., 2017). It can be seen from this that pilots may experience an increase in their psychological and physiological arousal and stress levels during more difficult flight tasks and larger flight maneuvering actions.

In order to mitigate the negative impact of human stress states on flight safety, it is essential to accurately identify and evaluate human stress states in the first place. At present, there have already been a number of studies, both domestically and internationally, carried out regarding stress assessment (Wang and Yang, 2024; Wang et al., 2021). In the book *Naval Aviation Medicine*, Li Minggao systematically elaborated on the stressors and stress responses of carrier-based aircraft pilots, as well as the relationships between these factors and flight performance (Li, 2015). Employing signal processing and machine learning techniques, in conjunction with a variety of physiological indicators, to detect and assess the stress states of individuals has emerged as one of the research approaches that have drawn significant academic attention (Khowaja et al., 2021). He used electrocardiogram indicators to conduct a binary classification of the stress levels of individuals under traditional mental arithmetic tasks, achieving an accuracy rate of 82.7% with a convolutional neural network model (He et al., 2019); Martinez used skin conductance and HRV indicators based on neural network methods to conduct binary classification of the stress levels of individuals under three different difficulty levels of 3D wooden puzzle tasks, achieving a maximum accuracy rate of 92.1% (Martinez et al., 2017).

Current research on stress encompasses multiple dimensions, including its definition, influencing factors, and physiological and psychological manifestations. Considerable exploration and analysis have been carried out regarding the patterns of physiological and psychological changes induced by stress. Nevertheless, the majority of existing studies predominantly concentrate on the medical health and automotive driving domains. In the aviation sector, research on stress, particularly with respect to pilots, remains relatively scarce. On the other hand, related studies have shown that integrating training on stressor coping mechanisms into the training of pilots can effectively improve pilots’ task performance under stress and alleviate the influence of pilot stress on the occurrence of aircraft accidents (McClernon et al., 2011). Therefore, conducting stress assessment research in the context of aviation operation tasks is conducive to rationally analyzing the intensity of stressors, enhancing pilots’ physiological and psychological capabilities to cope with stress, improving the stress support system, and intervening in post-stress effects, providing an important basis for the precise selection and training of pilots in the future.

When pilots are engaged in continuous and interactive control tasks, it has been found that α2 electroencephalogram (EEG), heart rate, blink rate, behavioral measurements, and subjective measurements are sensitive to changes in the load level of multiple tasks, while alpha event-related desynchronization (ERD) and theta event-related synchronization (ERS) indicators are not (Fournier et al., 1999). Skaramagkas V developed a robust emotional or cognitive computational model based on eye movement and pupil tracking-related indicators, which can detect visual attention, emotional arousal, and cognitive workload (Skaramagkas et al., 2023). However, currently, the commonly used and accessible indicators for in-flight physiological monitoring are electrocardiogram (ECG) and respiration, while behavioral data and eye movement data cannot be directly obtained during a pilot’s flight (Wang and Ma, 2024).

This study is based on the key event segments during flight, namely, the phases of aircraft maneuvers that may induce an increase in the pilots’ stress levels, to extract characteristic data from the pilots’ electrocardiogram and respiratory signals, and to conduct research on stress assessment indicators and stress assessment models for pilots. The research results are conducive to understanding the mechanism of changes in cognitive levels and operational capabilities of pilots under typical stress conditions, providing methodological tools for the effective monitoring and discriminant analysis of pilots’ stress states. This is beneficial for enhancing pilots’ stress response and handling capabilities, optimizing stress training strategies for pilots, and has significant theoretical and engineering application value for improving flight performance and ensuring flight safety.

## 2 Methods

### 2.1 The acquisition of experimental data

This study is based on a typical high-pressure task scenario of simulated air combat. Pilots were used as subjects, and a total of 32 flight data were collected during real flights. The data includes electrocardiogram signals, respiratory signals, and three-axis acceleration data of the human body.

#### 2.1.1 Experimental subjects

There are 32 pilots in a certain air force unit, all male, aged between 22 and 30, with flight hours ranging from 420 to 2,500. All the pilots are in good health and qualified for flight.

#### 2.1.2 Experimental methods

**FIGURE 1:**
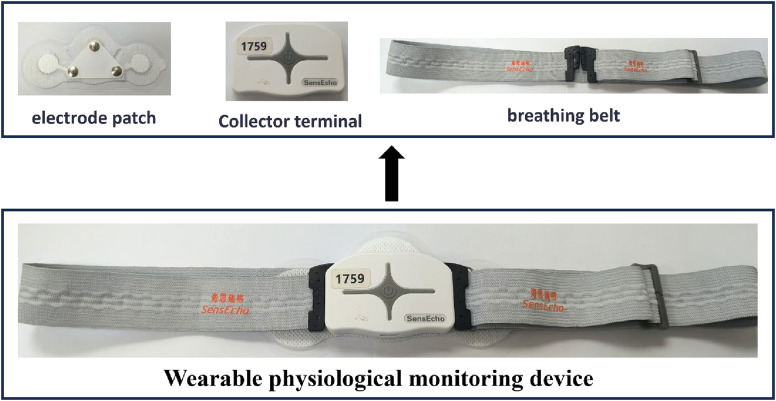
Composition of wearable physiological monitoring devices during flight.

Before boarding the aircraft, pilots wear wearable physiological monitoring devices to conduct in-flight physiological monitoring. The device mainly monitors electrocardiogram, respiration, and human body acceleration data, all of which are collected with a single lead. The equipment is produced by Beijing Haisirui Ge and its model is SensEcho-1A. This device consists of a collector terminal, a breathing belt and electrode stickers. The equipment composition is shown in Figure 1.

The electrode patches are installed at the bottom of the collector terminal. Remove the insulating film on the electrode patches and fix the collector below the chest. Then, put on the breathing belt. The breathing belt should naturally wrap around the chest of the person when worn. After wearing it, press and hold the device’s power button for 3 s to start recording electrocardiogram, respiratory and acceleration data, which will be stored in the device. After the flight, connect the computer to export the data. The data was exported as raw signal values via the DamicsDYC software for further processing and analysis in subsequent research (Shang et al., 2024).

### 2.2 Extraction of key event segments and establishment of stress labels

This study defines critical events as flight maneuvers that have a high probability of inducing stress in pilots during simulated flights, and critical event segments as the flight phases during which critical events persist. In this study, in combination with the flight mission design, the aircraft will perform multiple roll and circling maneuvers during combat-related flight missions, waiting for the attack opportunity and instructions. Based on the literature review and analysis, it was found that after performing offensive and defensive flight maneuvers, fighter pilots’ subjective stress perception and perceived exertion rate scores significantly increased, and their average heart rate significantly rose during the flight, indicating a high level of stress (Hormeño-Holgado and Clemente-Suárez, 2019). Therefore, in this study, the flight phases involving multiple barrel rolls and loops are marked as critical event segments that may induce high stress levels in the operators, while the flight phases involving basic maneuvers such as level flight are marked as non-critical event segments with relatively low stress levels for the operators.

**FIGURE 2:**
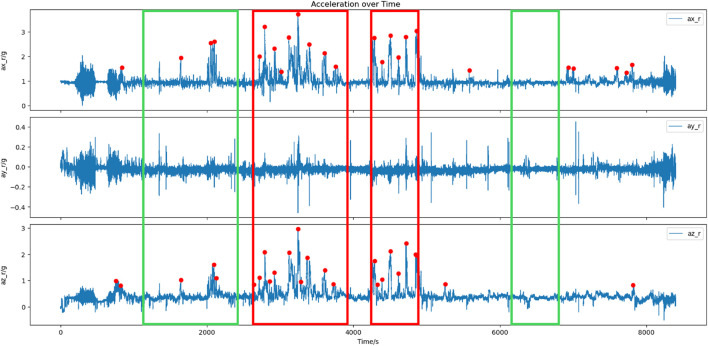
Illustration of the identified critical event segments and non-critical event segments in a certain flight (red represents critical event segments, and green represents non-critical event segments).

Take the acceleration data as the basis for identifying key events. When the acceleration in the horizontal or vertical direction exceeds the threshold range, it is regarded as a roll operation. If it exceeds the threshold range multiple times within a certain period of time, it is considered to have entered a critical event segment in the flight mission context. Specifically, in the process of extracting key event segments based on acceleration changes, when the algorithm detects that the acceleration in the x-axis, y-axis, and z-axis directions of the aircraft experiences multiple large fluctuations continuously, that is, multiple peaks appear successively, it is considered that the aircraft is in a multiple roll and turn segment. By identifying the peaks of acceleration changes during flight and taking five or more consecutive peaks as the identification standard for key event segments, the key event segments and non-key event segments identified in a certain flight are illustrated in Figure 2. In the flight data of 32 sorties, 42 critical event segments and 42 non-critical event segments were demarcated. These segments corresponded to the high-stress state and low-stress state of the pilots respectively, thus establishing the labels for the stress state assessment model.

### 2.3 Preprocessing of physiological signals and extraction of indicators

This study utilized the electrocardiogram (ECG) and respiratory indicators of pilots as the input for the stress state assessment model. The original ECG and respiratory signals were processed in sequence through phased extraction, signal preprocessing, and indicator extraction.

#### 2.3.1 Preprocessing of physiological signals

Given the long flight duration, large amount of data, and inconsistent durations of event segments in each flight, this study sliced the physiological signals under key/non-key event segments, with a slice duration of 3 min. The main basis for choosing the slice duration is: (1) Due to the need for extracting heart rate variability indicators from electrocardiogram data, existing research indicates that recording data exceeding 2 min can provide more reliable and accurate HRV analysis results (Wu et al., 2020). (2) Due to the demand for sample size in machine learning. Small sample data is prone to interference from abnormal data, leading to overfitting or poor generalization ability, which is insufficient to support the stability and reliability of the stress assessment model. (3) For the sake of ensuring data integrity, when slicing and extracting key event segments during the data processing, backward sampling is adopted to complete the last slice data to 3 min. Based on the above extraction principles, 608 physiological data sets of 3 min in length were extracted from 42 critical and non-critical event segments of 32 flight missions, with 304 data sets from each type of event segment.

Then, the signal was preprocessed based on the Neurokit2 library and the HeartPy library (Makowski et al., 2021; Van Gent et al., 2019). Signal processing includes noise cleaning and smoothing. The ECG signal is processed for baseline drift and filtering based on the preprocessing functions of the HeartPy library, while the respiratory signal is smoothed and filtered using the Neurokit2 library. The purpose of smoothing is to reduce noise and baseline drift, improving signal quality and interpretability; the purpose of noise cleaning is to filter out 50 Hz power frequency signals and high-frequency signal interference, thereby ensuring the reliability of subsequent index extraction.

#### 2.3.2 Extraction of physiological indicators

Based on the preprocessed ECG and respiratory signals, and referring to domestic and foreign research (Villafaina et al., 2021; Nemcova et al., 2021; Fiorini et al., 2020; Huysmans et al., 2018; Oskooei et al., 2021), this study uses the Neurokit2 library to extract 16 electrocardiogram (ECG) indicators and 5 respiratory indicators that are relatively sensitive to stress. The extracted electrocardiogram and respiratory indicators of stress sensitivity are shown in Table 1.

**TABLE 1:** Extracted electrocardiogram and respiratory indicators corresponding to stress sensitivity.

Indicator type	Indicator	Indicator meaning
Electrocardiogram indicators	Mean HR	The average heart rate
	Mean RR	The average value of RR intervals
	SDNN	The standard deviation of all RR intervals
	SDSD	The standard deviation of successive RR interval differences only represents short-term variations
	RMSSD	The root mean square of successive RR interval differences
	pNN20	The percentage of consecutive RR intervals with a difference exceeding 20 m
	pNN50	The percentage of consecutive RR intervals with a difference exceeding 50 m
	LF	The absolute power of the low-frequency band (0.04–0.15 Hz)
	LFn	Standardized low-frequency power is obtained by dividing the low-frequency power by the total power
	HF	The absolute power of the high-frequency band (0.15–0.4 Hz)
	HFn	The standardized high-frequency power is obtained by dividing the low-frequency power by the total power
	LF/HF	The ratio of low-frequency power to high-frequency power
	S	The elliptical area representing the total HRV is proportional to SD1 and SD2
	SD1	The standard deviation of the poincaré plot is perpendicular to the contour lines and serves as an indicator of short-term HRV variation, that is, the variability between heartbeats
	SD2	The standard deviation along the identity line in the poincaré plot is an indicator of long-term HRV changes
	SD1/SD2	The ratio of short-term to long-term changes in HRV.
Respiratory indicators	MeanRsp	The average value of respiratory rate
	EB1	The absolute power in the 0–0.1 Hz band
	EB2	The absolute power in the 0.1–0.2 Hz band
	EB3	The absolute power in the 0.2–0.3 Hz band
	EB4	The absolute power in the 0.3–0.4 Hz band

Existing research indicates that under stress, the sympathetic nervous system is activated, causing an accelerated heartbeat and an increase in blood supply to the body. After the stress is relieved, the parasympathetic nervous system will be activated, inhibiting the sympathetic nervous system and slowing down the heart rate. Under stress, time-domain indicators such as Mean HR tend to increase, while Mean RR, RMSSD, SDNN, and pNN50 tend to decrease; frequency-domain indicators such as LF and LF/HF tend to increase, while HF tends to decrease ((Kim et al., 2018; Järvelin-Pasanen et al., 2018; Yan et al., 2019).

### 2.4 Model construction and validation methods

This study employed machine learning methods, using electrocardiogram and respiratory indicators as model input features to discriminate the stress state of pilots. During the modeling process, several classic machine learning algorithms, including Decision Tree, Random Forest, and Extreme Gradient Boosting Tree, were respectively applied to train the basic models. During the training process, methods such as grid search and Bayesian optimization are used to determine the optimal parameters. By integrating learning algorithms to train combined models of basic models, the performance of the models is optimized. The five-fold cross-validation method is adopted to verify the model results, and the accuracy rate, precision rate, recall rate, and F1 score indicators are used to evaluate the performance of the models.

#### 2.4.1 Model structure

**FIGURE 3:**
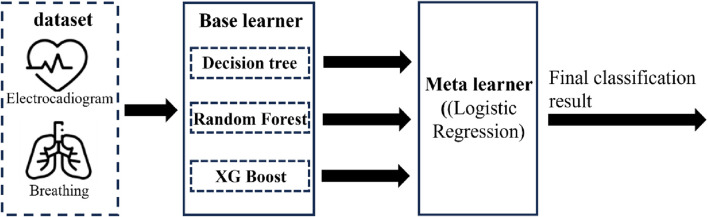
Overall construction thought of the model.

The overall construction idea of the model is shown in Figure 3. Firstly, basic models are trained by applying machine learning algorithms such as decision trees, random forests, and extreme gradient boosting trees. Then, ensemble learning algorithms are used to train the combined model of the basic models to further optimize the model performance.

1. The basic method of decision trees is to establish decision tree nodes based on features, split the data set according to the judgment of this feature, and recursively repeat the splitting until the pre-set termination conditions are met. The Gini coefficient is used to select the splitting feature to maximize information gain. The calculation formula is shown in Equation 1, where $p_{i}$ is the proportion of samples belonging to category i in the total samples, and n is the total number of samples.
$$Gini=1-\sum_{i=1}^{n}{p_{i}}^{2}$$ (1)
2. The base learner of random forest is decision tree. By training and classifying random samples in the dataset with multiple decision trees and combining the prediction results through weights or averages, the performance and generalization ability of the overall model are improved.
3. The core concept of Extreme Gradient Boosting (XGBoost) is to integrate numerous weak classifiers to yield a powerful classifier. XGBoost constructs a model by stacking multiple decision trees successively. At each layer, it endeavors to minimize the residuals of the decision tree from the previous layer, thereby enhancing the overall performance of the model gradually. In contrast to random forests, which randomly sample data, XGBoost is an additive model consisting of k base classifier models. Suppose the tree model trained in the t iteration is $f_{t}\left(x_{i}\right)$ . The predicted value ${\hat{y}}_{i}\left(t\right)$ of sample i after the t time iteration is expressed as Equation 2. Here, ${\hat{y}}_{i}\left(t-1\right)$ represents the predicted value obtained from the first t−1 trees.
$${\hat{y}}_{i}\left(t\right)=\sum_{k=1}^{t}f_{k}\left(x_{i}\right)={\hat{y}}_{i}\left(t-1\right)+f_{t}\left(x_{i}\right)$$ (2)
  XGBoost trains the boosting tree model by optimizing the objective function. The objective function $Obj\left(t\right)$ is shown in Equation 3, which consists of the loss function L and the regularization term Ω . The loss function represents the difference between the k time predicted value and the actual value, indicating the model’s bias. The regularization term is used to control the model’s complexity and prevent overfitting.
$$Obj\left(t\right)=\sum_{i=1}^{n}L\left(y_{\dot{i}},{\hat{y}}_{\dot{i}}\left(t-i\right)+f_{t}\left(x_{i}\right)\right)+\Omega \left(f_{t}\right)+C$$ (3)
  By approximating the loss function L through Taylor expansion, the gradient and second derivative of the objective function are optimized and calculated using gradient descent. Based on this information, the model parameters are updated, and the regularization term is used to prune the tree to obtain a new tree model. Through continuous iteration to correct the residuals, the optimal objective function is obtained.
4. The stacking generalization method (Stacking) is adopted to train a model for combining various base learners. Stacking first trains multiple different base learners, and then uses the output results of the base learners as features to train a meta-learner, thereby obtaining a final output. Through Stacking training, the different characteristics and advantages of the base learners can be fully exploited, thereby improving the overall performance of the model.

#### 2.4.2 Model validation methods

The performance of different models was evaluated using the 5-fold cross-validation method. The 5-fold cross-validation method divides the samples into 5 equal parts randomly. Each time, 4 parts are selected as the training set to train the model, and the remaining 1 part is used as the test set to test the model’s performance. A total of 5 rounds are conducted, with a different test set selected each time. Finally, the results of the 5 trained models are comprehensively evaluated.

The confusion matrix (Confusion Matrix) is an important tool for evaluating the performance of classification models, which can help understand the prediction effect of the model. The performance of the model is evaluated through accuracy, precision, recall, and F1 score metrics, the meanings of which are shown in Table 2.

**TABLE 2:** Model performance evaluation indicators.

Indicator	Definition	Formula for calculation
Accuracy	The proportion of samples predicted correctly by the model to the total number of samples characterizes the overall predictive performance of the model	$\frac{TP+TN}{TP+TN+FP+FN}$
Precision	The proportion of correctly predicted samples among those predicted as positive examples by the model characterizes the model’s prediction performance for positive examples	$\frac{TP}{TP+FP}$
Recall	The proportion of actual positive samples that the model successfully predicts as positive represents the coverage degree of the model for positive samples	$\frac{TP}{TP+FN}$
F1-score	The harmonic mean of precision and recall takes into account both the accuracy and recall ability of the model comprehensively	$\frac{2\times Precision\times Recall}{Precision+Recall}$

TP, stands for True Positive; TN, for True Negative; FP, for False Positive, and FN, for False Negative.

## 3 Result

### 3.1 Analysis of physiological index differences under different stress states

This study obtained 608 sets of data, each containing 16 electrocardiogram indicators and 5 respiratory indicators. Among them, there were 304 sets of data for the critical event segments and 304 sets for the non-critical event segments. High-dimensional input data may mask important indicator features, adversely affecting the accuracy of the analysis (Xue et al., 2022). Therefore, variance analysis is utilized to extract the indicators sensitive to stress and preliminarily screen the model input features. Statistical analysis was conducted using SPSS Statistics 26.0 software to perform a difference analysis on electrocardiogram and respiratory indicators under different stress levels. For variables that conform to the normal distribution and pass the homogeneity of variance test, an independent samples T - test was employed for analysis. For those variables that do not meet the requirements of the normal distribution or fail the homogeneity of variance test, Welch’s T - test was utilized for analysis.

Homogeneity of variance tests were conducted on the electrocardiogram indicators. The Mean HR, MeanRR, SDNN, RMSSD, SD2 indicators met the assumption of homogeneity of variance and were analyzed using the independent samples T-test. SDSD, pNN50, pNN20, LF, HF, LF/HF, LFn, HFn, SD1, SD1/SD2, S indicators do not meet the homogeneity of variance, and Welch’s T-test is adopted. The T - test results for all electrocardiogram indices are presented in Table 3. Among these, the Mean HR, MeanRR, SDNN, RMSSD, pNN50, pNN20, LF/HF, LFn, HFn, SD1/SD2 indicators exhibited statistically significant differences between the high - stress and low - stress levels. The SDSD and SD1 indicators showed marginally significant differences between high and low stress levels. The Mean HR, SDNN, LF/HF, LFn were significantly higher under high stress levels than under low stress levels. The MeanRR, RMSSD, pNN50, pNN20, HFn, SD1/SD2 were significantly lower under high stress levels than under low stress levels.

**TABLE 3:** Results of T-test for electrocardiogram indicators.

Indicator	Unit	T-test	P-value	Cohen’s d value
Mean HR	time/min	5.000	<0.001**	0.406
MeanRR	ms	−4.862	<0.001**	−0.394
SDNN	ms	2.173	0.030*	0.176
RMSSD	ms	−2.207	0.028*	−0.179
SDSD	ms	−1.884	**0.060**	−0.153
pNN50	%	−2.784	0.006**	−0.226
pNN20	%	−3.573	<0.001**	−0.290
LF	ms^2^	−1.300	0.194	−0.105
HF	ms^2^	−1.626	0.105	−0.132
LF/HF	—	5.058	<0.001**	0.410
LFn	Nu	4.992	<0.001**	0.405
HFn	Nu	−5.294	<0.001**	−0.429
SD1	ms	−1.884	**0.060**	−0.153
SD2	ms	−0.650	0.516	−0.053
SD1/SD2	—	−5.197	<0.001**	−0.422
S	—	−1.189	0.235	−0.096

**p < 0.01, *p < 0.05, no asterisk* in bold indicates marginally significant (0.05 < p < 0.1).

Homogeneity of variance tests were conducted for the respiratory indicators. The indicators of Mean Rsp met the assumption of homogeneity of variance, and independent samples T-tests were used; the indicators of EB1, EB2, EB3, EB4 did not meet the assumption of homogeneity of variance, and Welch’s T-tests were used. The results of the T-test for all respiratory indicators are shown in Table 4. Among them, the Mean Rsp, EB1, EB2, EB3, EB4 indicators showed significant differences between high and low stress levels, and were significantly lower at high stress levels than at low stress levels.

**TABLE 4:** Results of T-test for respiratory indicators.

Indicator	Unit	T-test	P-value	Cohen’s d value
Mean Rsp	time/min	−6.687	<0.001**	−0.542
EB1	ms^2^	9.213	<0.001**	0.747
EB2	ms^2^	7.561	<0.001**	0.613
EB3	ms^2^	6.645	<0.001**	0.539
EB4	ms^2^	5.876	<0.001**	0.477

*p < 0.05, **p < 0.01.

Bold indicates p < 0.1, representing marginally significant and significant. An asterisk(*) indicates p < 0.05, representing significant.

Based on the above results, a total of 10 electrocardiogram indicators and 5 respiratory indicators showed significant differences between the critical event period and the non-critical event period.

### 3.2 Results of the stress state assessment model

Take the 10 electrocardiogram indicators and 5respiratory indicators that show significant differences under different stress levels as the model inputs. The electrocardiogram indicators include: Mean HR, Mean RR, SDNN, RMSSD, pNN50, pNN20, LF/HF, LFn, HFn, SD1/SD2, and the respiratory indicators include: Mean Rsp, EB1, EB2, EB3, EB4. Models were constructed using decision tree, random forest, XGBoost and ensemble learning methods.

During the model construction process, the grid search method is utilized to determine the optimal parameters of the model. The decision tree model uses the Gini coefficient as the splitting criterion and selects the maximum tree depth as 3; the random forest model uses Shannon entropy as the splitting criterion, selects the maximum tree depth as 8, and the maximum number of iterations as 40; the XGBoost model has a learning rate of 0.12, a maximum tree depth of 7, a minimum sum of leaf node sample weights of 3, and an L1 regularization term parameter of 0.1. Decision tree, random forest, and XGBoost models are adopted as base learners, and logistic regression is used as the meta learner to construct an ensemble learning model.

**FIGURE 4:**
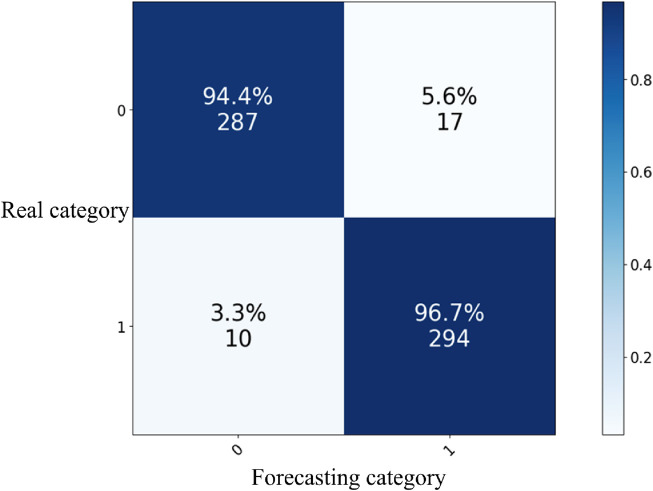
Confusion matrix of ensemble learning models with decision tree, random forest, and XGBoost as base learners.

Based on the above training process, the index results of the five-fold cross-validation of the model are shown in Table 5. Among them, the confusion matrix of the best-performing ensemble learning model is shown in Figure 4.

**TABLE 5:** Results of five-fold cross-validation for the model.

Model structure	Accuracy	Precision	Recall	F1 score
Decision tree	65.29%	61.96%	79.85%	69.44%
Random forest	71.38%	70.25%	74.64%	72.21%
XGBoost	71.22%	70.02%	73.89%	71.85%
Ensemble learning	**95.56%**	**94.53%**	**96.71%**	**95.61%**

Bold indicates p < 0.1, representing marginally significant and significant. An asterisk(*) indicates p < 0.05, representing significant.

The results show that the model performance is the best when Stacking optimization is carried out with decision tree, random forest and XGBoost as base learners. The accuracy rate reached 95.56%, with the precision rate for the two categories being 94.53%, the recall rate being 96.71%, and the F1 score being 95.61%. This effectively achieved the goal of distinguishing between high and low stress level samples, and significantly improved the performance compared to a single basic model.

## 4 Discussion

This study is based on flight data from a simulated air combat, a typical high-pressure task scenario. Key event segments were extracted from the acceleration data as stress labels. The Neurokit2 library was used to extract electrocardiogram and respiratory indicators from three perspectives: time domain, frequency domain, and nonlinearity. The analysis of variance method was employed to analyze the differences in physiological indicators under different stress states. Taking electrocardiogram and respiratory indicators that are more sensitive to stress states as model inputs, and using decision tree, random forest, and XGBoost as base learners, and logistic regression as the meta-learner, a binary classification model for pilots’ stress states based on electrocardiogram and respiratory features was constructed. The five-fold cross-validation accuracy rate of the model is 95.56%, thereby effectively achieving the assessment of pilots’ stress states based on physiological features.

In the analysis of differences in physiological indicators, the electrocardiogram indicators of Mean HR, Mean RR, SDNN, RMSSD, pNN50, pNN20, LF/HF, LFn, HFn, SD1/SD2, and the respiratory indicators of Mean Rsp, EB1, EB2, EB3, EB4 are sensitive to the stress levels of pilots and are key physiological features for the assessment and identification of their stress states. Among them, Mean HR, SDNN, LF/HF, LFn were significantly higher under high stress levels than under low stress levels, while Mean RR, RMSSD, pNN50, pNN20, HFn, SD1/SD2 were significantly lower under high stress levels than under low stress levels. This is consistent with the conclusion in existing studies that under stress, the indicators of Mean HR, LF, LF/HF will show an upward trend, while the indicators of Mean RR, RMSSD, SDNN, pNN50 will show a downward trend (Kim et al., 2018; Järvelin-Pasanen et al., 2018; Yan et al., 2019). The respiratory indicators that showed significant differences among different stress levels were all significantly lower at high stress levels than at low stress levels, which is in line with the respiratory indicators sensitive to stress proposed in existing literature (Shan et al., 2020). Therefore, the hypothesis proposed in this study that “the flight phases involving multiple barrel rolls and spiral maneuvers are identified as critical event segments that may induce high stress levels in the operators, while the flight phases involving basic maneuvers such as level flight are identified as non-event segments with relatively low stress levels for the operators” can be verified. The establishment of the stress labels in this study is relatively reliable.

During the model construction process, the classification accuracy rates of the decision tree, random forest, and XGBoost models were 65.29%, 71.38%, 71.22% respectively, and the classification effect was limited. Considering that in this study, data cleaning, standardization, and differential analysis were conducted prior to model construction, and the number of data points at high stress levels was equal to that at low stress levels. Moreover, during the model construction process, the grid search method and Bayesian optimization were employed for multiple parameter tuning. Thus, the impacts of factors such as poor data quality, inappropriate feature selection, sample imbalance, and insufficient parameter adjustment on the model were ruled out. The performance of the basic model is still not satisfactory, which might be due to getting stuck in a local optimum or being insensitive to certain characteristics of the data, thus failing to reach the global optimum. To address these issues, this study adopts an ensemble learning algorithm, combining multiple models to balance bias and variance and make up for the shortcomings of individual models. Compared with a single basic classification algorithm, ensemble learning algorithms can fully leverage the advantages and characteristics of different models to achieve better model performance and provide more accurate prediction results for the classification of pilots’ stress levels. The five-fold cross-validation accuracy rate of the ensemble learning model is 95.56%, not only outperforming the base model in terms of accuracy, but also demonstrating better class balance through an F1 score of 0.9561. Compared with similar studies, this research not only has reliable and authentic data sources that closely align with the pilot group, but also has further improved the classification accuracy rate (Martinez et al., 2017), achieving a relatively good two-classification assessment of pilots’ stress states based on physiological features.

However, this study still has certain limitations. In subsequent research, the generalization ability and reliability of the model can be enhanced by collecting more experimental data, expanding the training set through the method of extracting physiological features with a sliding window, or verifying the model’s ability to distinguish stress levels in other datasets. In addition, this study only established a binary classification model for stress. However, a more refined classification level is more valuable in practical applications. In subsequent research, a three-category label for stress states can be established, and an attempt can be made to build a three-classification model for stress to provide more accurate results for the assessment of pilots’ stress states.

## 5 Conclusion

Based on the typical high-pressure task scenario of air combat, this study collected electrocardiogram and respiratory data from 32 flights of pilots and conducted research on pilot stress assessment indicators and stress assessment models. The study employed an independent samples T-test to analyze the differences in the sensitivity of physiological indicators of pilots under different stress states. The results indicated that the Mean HR, MeanRR, SDNN, RMSSD, pNN50, pNN20, LF/HF, LFn, HFn, SD1/SD2 indicators in the electrocardiogram and the Mean Rsp, EB1, EB2, EB3, EB4 indicators in the respiratory system were sensitive to the stress levels of pilots, and they were the key physiological features for the assessment and discrimination of pilots’ stress states. Furthermore, a stress state assessment model was constructed using machine learning methods. The results indicated that the accuracy rate of the stress state assessment model built with the ensemble learning method in the five-fold cross-validation binary classification was 95.56%, effectively achieving the assessment of pilots’ stress states based on physiological features. The results of this study are conducive to providing methodological tools for the effective monitoring and discriminant analysis of pilots’ stress states, which is beneficial for optimizing pilots’ stress training strategies and enhancing their stress response and handling capabilities. This holds certain theoretical significance and engineering application value for improving flight mission performance and ensuring flight safety.

## Data Availability

The raw data supporting the conclusions of this article will be made available by the authors, without undue reservation.
